# Antimicrobial Peptides and Complement in Neonatal Hypoxia-Ischemia Induced Brain Damage

**DOI:** 10.3389/fimmu.2015.00056

**Published:** 2015-02-12

**Authors:** Eridan Rocha-Ferreira, Mariya Hristova

**Affiliations:** ^1^Perinatal Brain Repair Group, Department of Maternal and Fetal Medicine, Institute for Women’s Health, University College London, London, UK

**Keywords:** antimicrobial peptides, complement, neonatal hypoxia-ischemia, microglia, astrocyte, hypoxic-ischemic encephalopathy

## Abstract

Hypoxic-ischemic encephalopathy (HIE) is a clinical condition in the neonate, resulting from oxygen deprivation around the time of birth. HIE affects 1–5/1000 live births worldwide and is associated with the development of neurological deficits, including cerebral palsy, epilepsy, and cognitive disabilities. Even though the brain is considered as an immune-privileged site, it has innate and adaptive immune response and can produce complement (C) components and antimicrobial peptides (AMPs). Dysregulation of cerebral expression of AMPs and C can exacerbate or ameliorate the inflammatory response within the brain. Brain ischemia triggers a prolonged inflammatory response affecting the progression of injury and secondary energy failure and involves both innate and adaptive immune systems, including immune-competent and non-competent cells. Following injury to the central nervous system (CNS), including neonatal hypoxia-ischemia (HI), resident microglia, and astroglia are the main cells providing immune defense to the brain in a stimulus-dependent manner. They can express and secrete pro-inflammatory cytokines and therefore trigger prolonged inflammation, resulting in neurodegeneration. Microglial cells express and release a wide range of inflammation-associated molecules including several components of the complement system. Complement activation following neonatal HI injury has been reported to contribute to neurodegeneration. Astrocytes can significantly affect the immune response of the CNS under pathological conditions through production and release of pro-inflammatory cytokines and immunomodulatory AMPs. Astrocytes express β-defensins, which can chemoattract and promote maturation of dendritic cells (DC), and can also limit inflammation by controlling the viability of these same DC. This review will focus on the balance of complement components and AMPs within the CNS following neonatal HI injury and the effect of that balance on the subsequent brain damage.

## Introduction

Neonatal brain injury resulting from oxygen deprivation around the time of birth affects 1–3/1000 live term births in high-income countries with rates 5–10 times higher in low-resource setting. About 40% of the affected infants die in the neonatal period and additional 30% sustain lifelong neurological deficits, including cerebral palsy, epilepsy, and cognitive disabilities (1). Neonatal hypoxia-ischemia induces a robust inflammatory response in the immature brain, which is considered to play an important role in the development of brain damage and subsequent hypoxic-ischemic encephalopathy (HIE). Initial inflammation involves activation and recruitment of various immune cells into the injured brain. The initial pro-inflammatory response is followed by hypoxic-ischemic (HI) secondary energy failure that may last for days, followed by a switch to anti-inflammatory response and resolution. However, the exact mechanisms involved in the immune response following HIE still remain unknown. Several mediators of the inflammatory cascade include components of both innate and adaptive immune systems, such as cytokines, chemokines, adhesion molecules, as well as antimicrobial peptides (AMPs) and complement (C).

## Neonatal HI

Despite the neonatal period only constituting the first 28 days of life, it accounts for 38% of death in children younger than 5 years of age. Direct causes leading to neonatal death include infection (36%), prematurity (28%), and birth asphyxia (23%). The latter two, combined with congenital defects (7%) account for the majority of deaths occurring within the first week of life (2). This morbidity is generally a result of multiple organ dysfunctions (3) or termination of care. Epidemiological studies have shown that asphyxia is not the most common cause for developmental disorders; however, it poses important clinical problems, as infants who survive an asphyxia episode around the time of birth are at high risk of developing lifelong devastating impairments. Neonatal HIE and the ensuing clinical manifestation cause significant global public health burden (4), with infant sufferers at risk of subsequently developing cerebral palsy and/or other neurological dysfunctions such as cognitive impairment, epilepsy, and autism (4–6).

The pathophysiology of brain injury resulting from birth asphyxia includes evidence of fetal stress in the hours leading to birth, associated with depression at birth, need for resuscitation, evidence of metabolic acidosis as well as clinical and imaging signs of neurological anomalies (7). This phase is classified as primary energy failure, where reduction in cerebral blood flow and oxygen substrates leads to depletion in adenosine triphosphate ATP and phosphocreatine production and a switch from aerobic to anaerobic metabolism, causing accumulation of brain lactate and tissue acidosis (Table 1). Additionally, excitotoxic and oxidative cascades cause excessive stimulation of neurotransmitter receptors and cell membrane ionic transport failure, resulting in accumulation of intracellular calcium (8, 9), and successive cell swelling, activation of neuronal nitric oxide, and subsequent release of reactive oxygen species leading to mitochondria dysfunction, apoptosis, and programed cell death (10). As soon as the energy supplies are exhausted, cell necrosis occurs (11). Following successful reperfusion and resuscitation, there is a normalization of cellular metabolism and intracellular pH leading to neurotransmitter reuptake (12). However, in cases where the hypoxic-ischemic episode is severe or prolonged, these cascading events lead to a secondary energy metabolism failure in the mitochondria and subsequent persistence of excitotoxicity, oxidative stress, induction of inflammatory response, activation of caspase enzymes, and further apoptotic and necrotic cell death (12, 13).

**Table 1:** **Mechanisms of HI injury**.

**Primary energy failure**
Decline in cerebral blood flow, O_2_ substrates, and high-energy phosphate compounds
Initiation of neurotoxic cascade
Reduction of membrane homeostasis leading to calcium influx, mitochondrial dysfunction, brain acidosis, apoptosis, and necrosis
**Latent phase**
Normalization of oxidative metabolism
**Secondary energy failure**
Continuation of neurotoxic cascade
Inflammatory response
Caspase activation
Decrease in levels of protein synthesis and growth factors
Continuation of apoptosis and necrosis

## Neonatal HI and Inflammation

For a long period of time, the central nervous system (CNS) has been regarded as an immune-privileged site. The blood–brain barrier (BBB), formed by the endothelial lining of the cerebral capillaries, the arachnoid multi-layered epithelium, and the CSF-secreting choroid plexus epithelium, in conjunction with neighboring cell types such as astrocytes and pericytes, prevents infiltration of circulating immune cells, including B- and T-cells, and diminishes the influx of neurotoxic and neuroexcitatory agents from the blood flow (14). However, the CNS has the capacity to generate innate and adaptive immune response. In the CNS, the immune roles of peripheral neutrophils, dendritic cells (DC), macrophages, and natural killer cells are replaced by microglia, astrocytes, and oligodendrocyte precursors (15).

HI brain injury induces a robust inflammatory response in the immature brain (16). Furthermore, injury to neurons leads to a rapid change in their gene expression with stimulation of astrocytes and microglial activation and aggregation for survival support (17). Neuroglial activation is a graded response accompanied by secretion of pro-inflammatory cytokines, causing increased production of nitric oxide, reactive oxygen species, activation of the vascular endothelium, and recruitment of peripheral immune cells into the injured brain (18).

### CNS immune cells

#### Microglia

**Figure 1:**
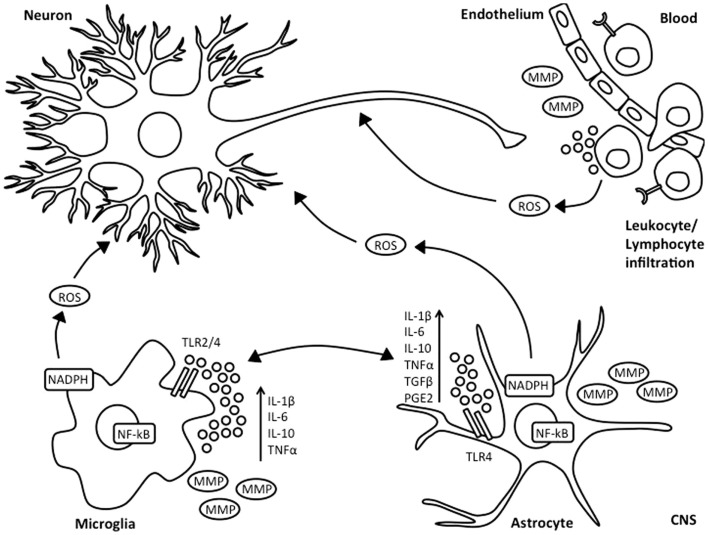
**Inflammatory response following HI injury**. Neonatal asphyxia leads to activation of microglia and astrocytes, which subsequently results in increased synthesis and secretion of pro- and anti-inflammatory cytokines, reactive oxygen species, and release of matrix metalloproteinases. This is associated with BBB breakdown, as well as influx of leukocyte and lymphocyte immune cells into the injured brain.

Microglia are considered as the resident macrophages of the CNS and account for 10–20% of total glial population. Under normal physiological conditions, microglia are present in a resting state with highly ramified and motile processes. However, in the presence of environmental changes to the brain, microglia become rapidly activated, undergoing morphological changes involving retraction of processes and increase in cell body size. Depending on the extent of damage, microglial cells will further activate, become phagocytic and migrate to the site of injury (19). Microglia play an important role in HIE. Retrospective post-mortem clinical studies have shown substantial microglial activation and infiltration in the hippocampal dentate gyrus of HIE infants, which was not observed in infants who had died from trauma or sepsis (20). Microglial contribution to secondary energy failure is thought to occur via production of pro- and anti-inflammatory cytokines such as interleukin (IL)-1β, IL-6, tumor necrosis factor-alpha (TNF-α) (21, 22), as well as expression of toll like receptors (TLRs) and antigen presentation (Figure 1). Microglial cells are also able to release matrix metalloproteinases, thus leading to breakdown of the BBB, allowing influx of leukocytes into the no longer immune-privileged CNS, thus exacerbating inflammation and subsequent brain damage (23). However, there are contradicting experimental mouse data on whether inhibition of microglial activation following neonatal HI is beneficial (24, 25). The microglial innate immune response is characterized by classical or M1 activation with subsequent production of associated pro-inflammatory molecules, followed by resolution and a switch to alternative or M2 phenotype leading to anti-inflammatory signaling and clearance of reactive species and wound healing (26).

#### Astrocytes

Astrocytes are the most abundant cell type in the CNS. They are essential supporters of brain homeostasis and neuronal function and also regulate synaptogenesis (27). However, post-mortem clinical studies have demonstrated a prevalence of astrogliosis of 15–40% within the white matter of HI infants (28). Under HI conditions, pro-inflammatory mediators, cytokines, and reactive species produced by damaged neurons and oligodendrocytes can lead to astrogliosis. Activated astroglia, despite not being considered as a traditional inflammatory cell, secrete inflammatory cytokines such as IL-1, IL-6, interferon-γ, and TNF-α (29). Increased levels of these cytokines exacerbate nitric oxide toxicity, and both apoptosis and necrosis, thus aggravating HI injury (30). Astrocytes can also produce chemokines, which attract migration of immune cells into the CNS (31). Astrocytes have TLRs and respond to TLR ligands. Following brain injury, astrocytes also express major histocompatibility complex (MHC) and co-stimulatory molecules, develop Th2 immune response, and inhibit expression of IL-12 (27).

### Peripheral immune cells

#### Neutrophils

It is well established that HI brain injury is associated with infiltration of inflammatory cells into the brain. Neutrophils are the most abundant type of leukocytes and are an integral part of the innate immune system. Although in adult rodent models of ischemic insult, neutrophils are known to accumulate within the brain as early as 4–6 h post injury, and lasting up to 48 h (32, 33), this does not appear to be the case in neonatal HI injury, where infiltration of neutrophils into the injured brain is less marked, with a lesser number present at 42 h post-insult. However, this same study demonstrated that neutropenic P7 rats had 70% reduction in brain swelling at 42 h post-HI when compared to littermate controls (34). Therefore, despite the suggestion that neutrophils do not accumulate in the immature brain following HI, they still play a relevant role in exacerbation of neonatal brain damage.

#### Lymphocytes

Lymphocytes are granulocyte blood cells crucial in the immune response, and contribute to either adaptive (B- and T-cells) or to innate (NK-cells) immunity. In experimental adult rodent studies, lymphocytes are shown to infiltrate the CNS within a few hours after cerebral ischemia, and to remain within the brain for several days (35, 36). An adult mouse study using RAG1^−/−^ mice deficient in both B- and T-cells has shown a substantial reduction in cerebral infarction in the mutants following cerebral arterial occlusion. Furthermore, the same study demonstrated that mature B-cell negative animals did not show an altered response to ischemic injury (37). Interestingly, in the neonatal mouse model of middle cerebral arterial occlusion (MCAO), T-cell infiltration appears to occur only after 24 h post-insult and persists for up to 96 after injury (38). This could be due to immaturity of lymphoid progenitors at this stage of brain developmental. A clinical study assessing peripheral blood if infants with HIE showed that blood mononuclear cells are still relatively undifferentiated in newborns, with reduced expression of surface markers (39). However, in chronic inflammatory response to HI injury, CD4 lymphocytes are present within the infarcted brain regions 7 days after injury, and persist within the area of damage for a long period of time (40).

#### Dendritic cells

Dendritic cells are antigen-presenting cells recognized by T-cells and acting as messengers between adaptive and innate immune system. Initial IL-1β and TNF-α response, as well as TLR activation cause translocation of NF-κB inflammation transcription factor into the nucleus of preferentially DC, and also macrophages and endothelial cells, inducing transcription of pentraxin-related protein (PTX3), a soluble pattern recognition receptor from the lectin family (41, 42). Pentraxin not only assists the recognition of microbes and amplification of innate immunity but is also involved in the clearing of self-components and decreased DC recognition of apoptotic cells (43). A study looking at global pattern of gene expression following neonatal HI has shown activation of PTX3, suggesting a possible role for DC involvement in the subsequent immuno-inflammatory response (16).

### Inflammatory mediators

#### Cytokines

Both pro- and anti-inflammatory cytokines and their receptors are present in the brain and cerebrospinal fluid, and act as an integral part of the CNS inflammatory response to adverse stimuli (44). In fact, it is widely accepted that cytokines work as a final common pathway to injury from a number of varying insults, including HI. The most widely study cytokines in ischemic models of brain injury are IL-1, IL-6, IL-10, TNF-α, and transforming growth factor-β (TGF-β). From these, IL-1, IL-6, and TNF-α appear to exacerbate brain injury (45), whereas IL-10 and TGF-β may have neuroprotective function following ischemic injury (46). The early response IL-1, IL-6, and TNF-α cytokines are believed to be influential in the progression of injury in the immature brain via stimulation of synthesis of other cytokines and adhesion molecules, and prompting leukocyte infiltration, which in turn will lead to further recruitment of immune cells, as well as induction of neuronal injury mediators such as nitric oxide. This continual and progressive stimulus has influencing modulatory effects on glial gene expression and activation. Depending on the extent of cytokine-mediated cytotoxic inflammatory cellular activation, cell damage and subsequent death occurs (47, 48). Prospective clinical studies have shown an association between high levels of IL-1, IL-6, and TNF-α and infants who are deceased at 1 year of age or diagnosed with cerebral palsy (49). Subsequent clinical studies have also demonstrated a correlation between IL-1 CSF levels and HIE (50). Serum IL-1β, IL-6, IL-8, and TNF-α have demonstrable correlation with the MRS biomarker of anaerobic respiration lactate/choline (51). Additionally, CSF IL-6 levels after neonatal asphyxia are also associated with both early and late neurological outcomes and severity in HIE (52).

#### Chemokines

Chemokines are chemotactic cytokines thought to act together with different adhesion molecules such as selectins, integrins, and immunoglobulins in order to control immune cell trafficking. These proteins play a detrimental role in various neurodegeneration models, including HI, ischemic stroke, and excitotoxic brain injury (53). A neonatal mouse study of HI injury has demonstrated that mRNA expression of chemokines precedes infiltration of immune cells into the brain, thus proving its relevance in the inflammatory response following insult to the immature brain (31, 40).

#### Adhesion molecules

Adhesion molecules, including selectins, integrins, and immunoglobulins, play an essential role in leukocyte infiltration to the brain. Initially, adhesion molecules have low affinity binding consisting of rolling of cells, resulting then in high affinity binding and firm adhesion (54). Targeting these molecules in stroke experimental models has demonstrated their importance in brain injury, as inhibition of leukocyte adhesion resulted in improved neurological and histological outcome, whereas over-expression increased tissue infarction (55–57). However, the role of adhesion molecules in HIE still remains largely unknown.

## Antimicrobial Peptides

Antimicrobial peptides are a diverse group of cationic polypeptides containing less than 100 amino acid residues. AMPs were discovered through studies of the insect antimicrobial defense mechanisms and the pathways involved in intracellular phagocytosis of bacteria in different mammalian species (58). For a long time, AMPs were associated with antimicrobial and antifungal activities through opsonization, agglutination, neutralization, or destruction of pathogens (59). Emerging evidence suggests chemotactic and immunomodulatory characteristics of AMPs through chemotaxis, phagocytosis, cytokine production, production of reactive oxygen species, and maturation of DC (59–61). Most AMPs have a positive charge and are divided into several categories based on primary structure and topologies (62), although the most well studied AMPs are cathelicidins and defensins.

Defensins contain six conserved Cys residues forming three disulfide bridges. Depending on the spacing between the Cys residues and the topology of the disulfide bonds defensins are classified in α-, β-, or θ- (61, 62). Defensins are present in many animal species and their expression is associated with cells and tissues involved in host defense against microbial infections (Table 2). Depending on the cell type expressing them, defensins act either intracellularly through oxygen-independent destruction of phagocytosed microorganisms or are secreted in the extracellular milieu where they directly attack the microbial membrane. Therefore, defensins are either stored as granules of neutrophils and Paneth cells of the small intestine or secreted by monocytes, macrophages, natural killer cells, keratinocytes, and epithelial cells (61).

**Table 2:** **Cell sources and expression of defensins and cathelicidin**.

Name	Defensin type	Cell source	Tissue	Production	Activity
HNP 1–4	α	PMNs	Abundant	Constitutive	Antimicrobial
				Inducible	Antiviral
HBD 5–6	α	Paneth cells	Abundant	Constitutive	Antimicrobial
		Epithelial cells			Chemotactic for PMNs and T-cells
HBD-1	β	Epithelial cells	Urinary and respiratory tracts	Constitutive and inducible (LPS, peptidoglycan, interferon-γ)	Antimicrobial
		Keratinocytes			Chemotactic for monocytes, dendritic cells, and CD4 T-cells
HBD 2–4	β	Epithelial cells	Psoriatic scales	Inducible (IL-1, TNF-α, LPS)	Antimicrobial
		Keratinocytes	
LL-37	Cathelicidin	Epithelial cells, neutrophils, T- and B-lymphocytes, NK-cells, keratinocytes	Thymus, spleen, skin, liver, bone marrow, stomach, intestine and testis	Constitutive and inducible (insulin-like growth factor 1, TNF-α, IL-1 α, IL-6)	Chemotactic for granulocytes and CD4 T-cells

*HNP, human neutrophil peptide; HBD, human beta defensin; PMN, polymorphonuclear leukocytes*.

α-defensins were first characterized as antimicrobial proteins purified from extracts of cytoplasmic granules of polymorphonuclear leukocytes (PMNs) (63). Human α-defensins are produced by leukocytes, Paneth cells, and epithelial cells of the female urogenital tract. There are six α-defensins, called human neutrophil peptide (HNP) 1–4 and human defensins 5–6 (64). In addition to their antimicrobial activity, some α-defensins (HNP-1) possess also antiviral characteristics. HNP-1 inhibits HIV and influenza virus replication, and inactivates herpes simplex virus, cytomegalovirus, vesicular stomatitis virus, and adenovirus (61).

There are four β-defensins known as human beta defensins (HBDs) 1–4 and possessing structural similarity to the α-defensins. HBD-1 was first isolated from human plasma and is constitutively synthesized by epithelial cells of the urinary and respiratory tracts (62), as well as keratinocytes (65). HBD-1 expression can be up-regulated through treatment with lipopolysaccharide (LPS), peptidoglycan, and interferon-γ (62). HBD-2 was first purified from psoriatic scales and its expression overlaps with that of HBD-1, but HBD-2 is also present in skin, pancreas, leukocytes, and bone marrow. HBD-3 was identified simultaneously in psoriatic scales and through bioinformatics, and apart from epithelia is also expressed at lower levels in non-epithelial cells of the heart, liver, fetal thymus, and placenta. HBD-4 was identified by genomics (66) and its expression has been assessed through detection of mRNA and considered to occur primarily in testis and epididymis (62). HBD2–4 are inducible and can be up-regulated in response to pro-inflammatory stimuli such as IL-1, TNF-α, and LPS. Multiple defensin genes have been discovered suggesting more HBDs on peptide level (67).

The third family of defensins, the θ-defensins, generate from precursor peptides of α-defensins (68) and have been identified in rhesus macaque monkey leukocytes. The θ-defensins are not expressed in humans due to mutations encoding premature stop codons (69).

Cathelicidins are another major group of structurally and evolutionary distinctive mammalian AMPs constitutively expressed in thymus, spleen, skin, liver, bone marrow, stomach, intestine, and testis, and therefore similar in abundance of expression to the defensins (61, 66, 68). There is only one human cathelicidin gene encoding the amphipathic alpha-helical peptide LL-37 (64). Cathelicidins are constitutively expressed in epithelial cells, neutrophils, T- and B-lymphocytes, NK-cells, and in mouse and human mast cells, and their synthesis can be enhanced by LPS and lipoteichoic acid (66, 70). Cathelicidins have direct antimicrobial effect on Gram+ and Gram− bacteria and their synthesis in keratinocytes can be induced by *Staphylococcus aureus*. Some cytokines (insulin-like growth factor 1, TNF-α, IL-1α, and IL-6) can also induce the synthesis of LL-37 in keratinocytes (61). There are other AMPs, i.e., lysozyme, azurocidin, and bactericidal/permeability-increasing protein, which also possess antimicrobial activities and enhance phagocytosis (64).

Although AMPs are mostly known for their anti-bacterial properties, a great number of them also possess chemotactic features. α-defensins are chemotactic for PMNs and T-cells, HBDs for monocytes, DC, and CD4 T-cells, while LL-37 are chemotactic for granulocytes, as well as CD4 T-cells. All this suggests an essential role of AMPs as a link between innate and adaptive immunity. Generally, AMPs have antimicrobial properties, but are also an essential part of the inflammatory response (71), and different environmental stimuli involving multiple signaling pathways promote their synthesis. Pro-inflammatory molecules (IL-1, TNF-α, IL-6) and bacterial products augment the expression of cathelicidins and defensins through activation of AP-1, JAK2, and STAT3 signaling pathways (61). Altogether AMPs appear to be a crucial component of the antimicrobial host defense, directly inactivating the pathogens and contributing to the immune response associated with the pathogen removal.

## Complement

The complement system is a crucial component of innate immunity and is responsible for the recognition and elimination of pathogens. Its activation is associated with inflammatory mediation (72, 73) and induction of pro-inflammatory cytokines secretion (72). Activation of the complement system also facilitates clearance of toxic cell debris and apoptotic cells (74–76), as well as immune complexes (72, 76, 77).

The complement system plays an important role in various inflammatory disorders. Its activation can significantly contribute to inflammation-mediated tissue damage following ischemic-reperfusion injury (78), whereas complement deficiencies highly favor the development of autoimmunity (79). The accumulation or unsuccessful removal of cellular debris may contribute to autoimmune disorders like systemic lupus erythematosus (75), as well as various chronic inflammatory diseases like age-related macular degeneration (76), rheumatoid arthritis (80), and asthma (81).

**Figure 2:**
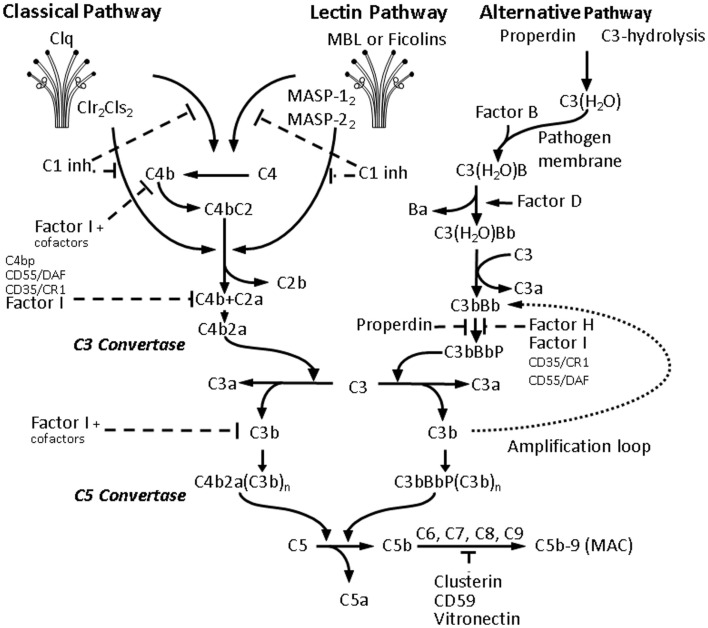
**Activation of the complement cascade**. Activation of all three C pathways generates homologous variants of C3-convertase cleaving C3 into C3a and C3b, whereas C3a stimulates mast cell degranulation and has chemotactic properties, and C3b acts as an opsonin and binds to the surface of pathogens. Increasing C3b deposition leads to the formation of C5-convertases cleaving C5 into the chemotactic C5a, and the fragment C5b, which together with C6, C7, C8, and the polymeric C9 forms the membrane attack complex (MAC) leading to the formation of transmembrane channel and osmotic lysis of the targeted pathogen. The classical pathway (CP) is initiated by binding of the C1-complex, consisting of a C1q molecule and a tetramer of 2 C1r and 2 C1s molecules, to antigen-bound IgM or IgG. The C1-complex cleaves C2 and C4 into C2a and C2b, and C4a and C4b, respectively. The C2a and C4b fragments form the CP C3-convertase. The lectin pathway activation is due to binding of mannose-binding lectin (MBL) and ficolins (Ficolin-1, -2, and -3) to carbohydrate pattern on microorganisms and dying cells, thus activating the MBL-associated serine proteases MASP-1 and MASP-2, which would in turn cleave C2 and C4. The alternative pathway (AP) is continuously activated through spontaneous C3-hydrolysis, resulting in formation of C3 convertases, which cleave C3 to a C3b-like C3, i.e., C3(H_2_O). Complement regulators are typically present on host cells and absent on pathogens, thus allowing C3(H_2_O) to bind factor B on the surface of the latter, and form additional C3 convertases after activation by factor D. In the presence of Factor D, C3(H_2_O)B is cleaved to Ba and Bb and forms C3(H_2_O)Bb, which in turn cleaves C3 to C3a and C3b forming C3bBb, which is stabilized by properdin. Properdin bound to microbial surfaces and apoptotic and/or necrotic cells can recruit C3 and also activate the AP (82). The final C3bBbP complex enzymatically cleaves more C3 and amplifies C activation. The C3-convertase of the AP can bind another C3b fragment and the resulting complex C3bBbP(C3b)_n_ acts as a C5-convertase and triggers the formation of MAC and pathogen elimination.

The complement system consists of more than 30 soluble and cell-associated factors and can be activated through three pathways (classical, alternative, and lectin) (Figure 2). The components of the complement system are synthesized to a great extent not only by hepatocytes but also by tissue macrophages, blood monocytes, and epithelial cells of the gastrointestinal and genitourinary tracts.

## Innate Immunity of the Brain

Both AMPs and C components are important factors of the innate immune system. Besides the fact that AMPs and C components are mostly produced in the periphery and that the BBB permeability is not absolute, there is evidence suggesting that both groups of proteins can be also produced in the brain (15, 83, 84).

### Glial cells in innate immunity of the brain

As previously mentioned, both microglia and astroglia are important participants in the innate immune response of the brain and both cell types can produce complement components and AMPs, as well as cytokines (Table 3).

**Table 3:** **Expression of TLRs, complement components, and antimicrobial peptides by microglia and astrocytes**.

Cell type	TLRs	C components	C regulators	C receptors	AMPs
Microglia	TLR1, TLR3, TLR5-9 (158)	C1q, C1r, C1s, C2, C3, C4 (73)	C1-inhibitor (73)	C1qR, CR3, C3aR, CR4, C5aR (73)	HBD-1(109)
	TLR2 (159)		CD59, CR1 (15)		LL-37 (91)
	TLR4 (160)	
Astrocytes	TLR2 (161, 162)	C1q, C1r, C1s, C2, C3, C4, Factor B, Factor D, C5–C9 (73)	C1-inhibitor, Factor H, Factor I, S protein, clusterin (73)CD59, DAF, MCP, CR1 (15)	C1qR, CR2, C3aR, C5aR (73)	HBD-1, HBD-2 (83), LL-37 (91)
	TLR3 (163)			
	TLR4 (162, 164)	
	TLR5 (162, 164)	
	TLR9 (162, 164)	

### Neurons in innate immunity of the brain

In the brain, immune function and modulatory activity are not features attributed only to immune competent cells, i.e., microglia and astrocytes, but also to non-immune cells. Neurons were originally considered to be just effector cells of C activation and neurodegeneration resulting from glial activation or cytokine influx through the BBB. However, neuronal expression of mRNA for C1q, C2, C3, C4, C5, C6, C7, C8, and C9 has been observed in post-mortem tissue from patients with Alzheimer’s disease (AD) (15, 73). Neuronal expression of C1-inhibitor has also been registered in AD cerebral tissue, suggesting expression of C regulator proteins and protection from full C activation associated with membrane attack complex (MAC) formation and cell lysis. Clusterin, C3aR, Factor H, and S protein have also been detected in neurons (73). Therefore, through its capacity of *de novo* synthesis of C components and regulators, the neuronal population appears to be an active player in the innate response of the CNS. So far, there is no data suggesting neuronal AMP production.

### Oligodendrocytes in innate immunity of the brain

Oligodendrocytes have also been shown to express C components like C3, as well as C regulator proteins, in particular C1-inhibitor, Factor H, S protein, and clusterin (73).

### Endothelial cells in innate immunity of the brain

Although the data suggesting expression of C components by cerebral epithelium is quite limited and points only toward production of C3 (85) peripheral endothelium has been proven to synthesize C1, Factor B, Factor H, and C5aR. Therefore, there is a possibility that brain epithelium might be also producing these C components. In respect to production of AMPs, synthesis and expression of HBD-2 mRNA and protein have been observed in human brain capillary endothelial cells following exposure to *Chlamydophila pneumoniae* (86). Overall, this data suggest a potential role of brain epithelium in innate immune response and modulation.

## Complement Components and AMP Expression in the Brain Under Normal Conditions

Some immune proteins such as pro-inflammatory cytokines (TNF-α, IL-6), MHC 1, and MHC receptors, apart from their capacity to trigger and participate in an immune response, also possess non-immune characteristics. Since C components are shown to similarly demonstrate non-immune features, for example, promote proliferation and regeneration in peripheral tissues (76), it is possible that they also execute analogous functions in the CNS. This hypothesis is supported by the observation that C3aR can regulate *in vitro* differentiation and migration of neural progenitor cells (87). In a study looking at the capacity of the classical C pathway to mediate CNS synapse elimination, Stevens and colleagues observed association of C1q and C3 with remodeling of synaptic connections in the visual system of the developing mouse brain (88). Chu and colleagues observed enhanced synaptic connectivity and epilepsy as a result of global deletion of C1q in mice (89). C1q also augments microglial clearance of apoptotic neurons and neuronal blebs and modulates the subsequent inflammatory cytokine production (90).

In the CNS, only HBD-1 is proven to be constitutively expressed. Hao and colleagues detected mRNA for HBD-1 in cultured microglia, astrocytes, and meningeal fibroblasts, but not in neurons (83). Conversely, HBD-2 expression is not constitutive but inducible and can be detected following exposure to LPS and/or pro-inflammatory cytokines (LT-1β, TNF-α) (83). The expression of cathelicidin LL-37 is also inducible and reported in cerebrospinal fluid and serum from patients with bacterial meningitis (91).

## Complement Components and AMP Expression in the Brain Under Pathological Conditions

Most of our knowledge of the expression and function of C components and AMPs in the brain is derived from studies of different brain diseases. Both C and AMPs have been registered in bacterial meningitis and cerebral infections, in trauma, stroke, and reperfusion injuries, as well as chronic conditions of brain injury such as AD, multiple sclerosis (MS), Parkinson’s, and Huntington’s diseases.

### Complement and AMPs in Alzheimer’s disease

Cribbs and colleagues observed up-regulation of innate immune system pathways in post-mortem hippocampus from aged and AD patients (92). The C system is associated with the inflammatory response, occurring around the neurofibrillary tangles and amyloid-β (Aβ) plaques in AD. Interestingly, different expression of C components is associated with the different neuropathological progression stages of AD (15). In the early stages of AD, C1q, C4d, and C3d are found, but MAC is absent, while in later stages the levels of C1q, C4d, and C3d are more prominent and MAC is registered in neurofibrillary plaques and neurite tangles (93–98). Yasojima and colleagues observed increased level of C1q in entorhinal cortex, hippocampus, and mid-temporal gyrus, characterized with high density of Aβ-plaques and neurite tangles (99). Additionally, Tooyama and colleagues demonstrated that C1q in the Aβ plaques is endogenously produced in the AD brain (100) suggesting C1q as an important mediator of AD inflammation. Genome wide association studies have allowed considerable progress in understanding AD genetics, identifying loci, including CR1, which are significantly associated with AD susceptibility (101–103).

The Aβ-plaques and the neurofibrillary tangles in AD have been mostly associated with classical C pathway activation, whereas alternative pathway (AP) has been documented only in the Aβ-plaques in human AD patients (104), and in murine AD models (94). The participation of the AP in AD inflammation has been confirmed through AD mouse model using C1q^−/−^ mice, where products resulting from C3 cleavage and properdin were registered in the Aβ-plaques (94, 105). Fonseca and colleagues demonstrated that treatment with PMX205, a C5aR antagonist, significantly reduces neuropathology in a mouse model of AD (106). However, the data retrieved from mouse models of AD and associated with complement activation should be cautiously considered due to the differences between the mouse models. Fonseca and colleagues observed a much slower progression of the disease in 3xTg mice compared to other transgenic strains and suggested AP activation or a C3-independent cleavage of C5 accounting for the detrimental outcome in these mice (107).

Conversely, the C system might as well play a protective role in AD (93). Osaka and colleagues have demonstrated that C5a may protect against excitotoxicity and activate neuroprotective mitogen activated protein kinase (108).

The data referring to the role of AMPs in AD are relatively limited, although the inflammatory process occurring in AD is associated with increased levels of HBD-1 mRNA in choroid plexus epithelium and HBD-1 protein in hippocampal neurons (109). Therefore, the nature of the AD inflammatory response is complicated and involves both C system and AMPs.

### Complement and AMPs in multiple sclerosis

C activation in MS is lesion and location dependent. In white matter lesions, C3d and C4d are detected and most likely covalently bound to myelin sheaths, while C3d, C1q, and C5 are associate with disrupted myelin, micro- and astroglia, and vessel walls (15, 110, 111). It is possible that some C factors in the white matter lesions are rapidly turned over as detection of C1q and MAC on myelin sheaths so far has not been successful (15). In gray matter lesions, C activation is very low (110), while in mixed white and gray matter C3d and C4d are detected on the myelin sheaths on the border of the lesions and C3d is only registered in the blood vessels (15). The production of C factors in MS is most likely endogenous, with macrophages considered as a main source of C1q and C3 and astroglia testing positive for C components in all lesions.

Direct link between AMPs and MS has not been yet established. Ultraviolet-B irradiation and vitamin D are important factors explaining the geographic variation and the increased prevalence of MS in areas with lower amount of sunshine (112). Once MS has developed, ultraviolet-B irradiation and vitamin D can reduce the severity of the disease through vitamin D-induced apoptosis of CD4 T lymphocytes (113). Vitamin D enhances innate immunity and the transcription of cathelicidin (114) and different defensins (115). Therefore, the balance of AMPs might be an important factor associated with the control of the inflammatory process in MS.

### Complement and AMPs in stroke and reperfusion injuries

The pathobiology of stroke involves an inflammatory response associated with all stages of the ischemic cascade, starting from the early damaging events triggered by arterial occlusion to the late regenerative processes underlying post-ischemic tissue repair (23). Increased immunoreactivity for C1q, C3c, C4d, and C9, and virtually absent C regulators were registered in ischemic lesions from patients with acute brain ischemia or ischemic stroke, suggesting activation of the classical C pathway (116). Therefore, the combination of increased deposition of C components and decreased expression of C regulators is a possible mechanism of tissue damage during ischemia in human brain. Supporting evidence for this hypothesis is provided by the study of Van Beek and colleagues who characterized the expression of different C components following permanent MCAO in the mouse (117). Their data demonstrate increased levels of C1q and C4 mRNA in ischemic cortex and increased expression of C4 in perifocal neurons suggesting local expression of C components, which (i) may contribute to the inflammatory process and represent a key component in secondary injury and (ii) may result in the formation of MAC and contribute to host cell lysis (117). The role of the AP in ischemic stroke has not yet been fully investigated, although a study by Elvington and colleagues in a murine model of MCAO suggests that the AP propagates cerebral inflammation and injury through amplification of the complement cascade (118).

Conversely, some data suggest protective role of the C system following ischemic injury, proposing that C activation does not appear to be a primary contributor to brain injury in a rabbit model of acute thromboembolic stroke (119) and also that C activation contributes to remodeling during repair in the CNS (73).

There is no data directly connecting AMP expression and stroke, although the inflammatory response resulting from ischemic injury can be associated with AMP production. Williams and colleagues proposed that central to the innate and adaptive immune response, and the prolongation of inflammation within the brain, is a dysregulation of constitutively expressed and inducible AMPs (84). *In vitro* exposure of human primary epithelial cells to high levels of glucose or low insulin results in decreased expression of HBD-2 and HBD-3 (84). Ischemic events in the adult brain are associated with the occurrence of chronic hyperglycemia, which can contribute to glycation of specific amino acid residues on AMPs, resulting in conformational changes and inhibition or prolongation of AMP function (84, 120). Therefore, the balance of AMP expression in the brain might be crucial for the inflammatory processes and subsequent occurrence of brain damage in the CNS.

## Complement and Neonatal HI

Complement is an essential aspect of innate immunity, and plays a role not only in normal brain physiology but also during pathology, including ischemia. Experimental research using rodent models of HI are now starting to clarify its role in hypoxic-ischemic brain injury.

A hallmark of hypoxia-ischemia primary energy failure is acidosis. A study by Sonntag and colleagues looking at umbilical arterial pH 22–28 h after birth has shown that serum C3a and C5a are increased after fetal acidosis (121). Another clinical study has demonstrated that circulating C3 is reduced following neonatal asphyxia (122). Initial experimental rodent studies have shown that C9 administration appeared to be detrimental (123), and that cobra venom factor (CVF) treatment did not affect HI induced brain injury in a study by Lassiter et al. (124). However, this same treatment approach was performed subsequently by Cowell and colleagues, and shown that CVF pretreatment decreases brain infarction following neonatal HI (125). Precise studies using C1q knockout mice were used to investigate the classical C pathway role in neonatal HI. A study by Ten and colleagues revealed that C1q^−/−^ mice had substantial reduction in brain infarction, as well as neurofunctional impairment when compared to wild type controls. Furthermore, wild type mice demonstrated greater deposits of C1q and C3 deposits within the brain as well as the presence of granulocytes in the area of infarction (126). This study strongly suggests that classical complement activation and subsequent brain deposition of C1q and C3 is not only associated with infiltration of granulocytes but also with HI brain injury. This hypothesis was further strengthened when the same group looked at brain mitochondria, and demonstrated that neurons of C1q^−/−^ mice were resistant to hypoxia-ischemia, with preserved brain mitochondria respiration, and reduced production of reactive oxygen species. Additionally, this study demonstrated that classical complement activation detrimental role in hypoxic-ischemic injury does not involve activation of MAC (127).

C3 is expressed in the brain by both neurons and glial cells (Table 3). Its activation and subsequent generation of C3a is known to have pro-inflammatory properties, and its expression appears to be detrimental in several models of CNS injury. However, C3a also has anti-inflammatory properties following LPS administration, by decreasing LPS-mediated cytokine release (128). Additionally, *in vitro* studies have shown its neuroprotective effects by acting on both microglia (129) and astrocytes (130). C3a can bind to its canonical receptor C3aR, and according to some controversial data to the alternative receptor C5L2 (131–133), which is expressed in both neurons and glia cells and has anti-inflammatory properties (134). However, in a study by Järlestedt and colleagues, it was demonstrated that canonical over-expression of C3a in astrocytes resulted in reduction of HI-induction of hippocampal tissue loss, as well as reduced numbers of astrocytes and microglia/macrophages in the ipsilateral striatum, suggesting that C3a protective role following HI is a result of its binding to C3aR (135).

Overall, experimental studies have shown that C1q is highly present in the brain following ischemia (136), and that classical complement pathway activation via C1q generates C3a and C5a pro-inflammatory mediators, which are associated with HI brain injury (137, 138), as well as complement-associated genes (16). Additionally, deletion of C1q not only reduces brain infarction and neurofunctional deficit but also results in protection of mitochondria respiration, indicating a role for classical complement activation and brain oxidative stress (127), and demonstrating a link between innate immunity and oxidative stress. Conversely, C3aR-mediated activation of C3a has also revealed a degree of protection following HI insult.

## AMPs and Neonatal HI

Currently, there is no direct evidence for the involvement of AMPs in neonatal HI brain damage. In respect of the capability of the CNS to locally produce AMPs and the evidence for their participation in inflammation-associated diseases such as AD and MS, AMP involvement in neonatal HI inflammation and subsequent brain damage is quite possible.

As previously described neonatal HI triggers an inflammatory response including activation of microglia, astroglia, DC, and is associated with release of pro- and anti-inflammatory cytokines, chemokines, and adhesion molecules. According to Bain and colleagues one of the reasons for the occurrence of HI brain damage is the misbalance in the release of pro- (IL-1, IL-6, IL-8, TNF-α) and anti-inflammatory (IL-10) cytokines promoting differentiation of oligodendrocyte precursor cells into astrocytes, but not oligodendroglia thus impairing subsequent myelination (139). In a mouse model of neonatal HI, Shrivastava and colleagues observed up-regulation of pro-inflammatory IL-1β, IL-6, and TNF-α and modulation of anti-inflammatory cytokines IL-1 receptor antagonist, IL-4, IL-13, and IL-10 (140). The up-regulation of IL-1β is to a great extent due to microglial activation in response to the HI injury and subsequently affects astrogliosis. Both IL-1 and IL-6 have been implicated in the induction and modulation of reactive astrogliosis (141). Microglial IL-1β might be directly affecting astroglial activation (142), while the effects of IL-6 on astrogliosis might be either direct or through the JAK2/STAT3 pathway as STAT3 is a critical transcription factor regulating astroglial maturation and GFAP expression (143, 144). Activated astroglia can subsequently produce HBD-2 (83) and alter the innate immune response in the brain following neonatal HI injury.

Neonatal HI might be directly affecting astrocytes promoting IL-1β (142) production, which can amplify astroglial and microglial activation and stimulate both cell types to produce AMPs. Conversely, activated astrocytes can also down regulate microglial activation through production of anti-inflammatory cytokines such as transforming growth factor β and prostaglandin E_2_ (145, 146) thus limiting inflammation and subsequent neurodegeneration (142). This can potentially affect microglial production of AMPs.

Post-HI hyperglycemia is harmful for the HIE (147). Although results obtained from adult experiments cannot be directly transferred and used as explanation for neonatal data due to differences in the level of maturation and enzyme development (5, 140), some pathways might be valid in adult as well as neonatal set-up. Therefore, observations of low AMP levels due to hyperglycemia and/or increased insulin resistance associated with many neuropathologies such as AD (84) might be also valid following neonatal HI. Reduction of mRNA for HBD-2 and HBD-3 has been observed after *in vitro* exposure of human epithelial cells to high glucose and/or low insulin (84). Therefore, the detrimental effects of hyperglycemia following neonatal HI might be attributed to alteration of AMP production.

Toll like receptors are important for antigen recognition in the CNS. TLR-regulated responses can control elimination of cell debris and promote repair in the brain (148) and are suggested to play an important part in CNS inflammatory conditions, including ischemia (141, 149). Microglial cells express TLRs and respond to TLR ligands (Figure 1) (148). TLR-4 is expressed by astrocytes, endothelial cells, and neurons (150, 151). Hao and colleagues suggested that astrocytic production of HBD-2 depends on cytokine stimulation of TLR, IL-1β, and TNF-α receptors (83). The precise mechanism through which astrocytes produce defensins is unclear, but TLRs induce NF-κB activation in response to cytokines and bacterial toxins, thus stimulating astrocytes to produce AMPs (64) (Figure 1). This hypothesis might be also valid in respect to astrocytic AMP production following neonatal HI.

Another mechanism through which AMPs might be affecting the inflammatory response following neonatal HI is through their ability to recruit immature DC to the site of inflammation and promote DC maturation through TLR-4, thus modulating the adaptive immune response of the brain (152).

All these suggest that misbalance of constitutively expressed (HBD-1) and inducible (HBD-2, HBD-3) AMPs, as well as cathelicidin (LL-37) is likely to occur in cases of inflammation-associated neurodegeneration in the adult brain, as well as following neonatal HI.

So far, there is no evidence suggesting direct damaging effects of AMPs on mammalian cells (83). Supportive evidence for the role of AMPs in the innate immune response following neonatal HI is the use of novel innate defense regulator peptides (IDRs) in animal models of neonatal cerebral inflammation and injury. IDRs are synthetic derivatives of endogenous cationic host defense peptides such as cathelicidin, selectively suppressing inflammation and augmenting protective immunity to pathogens (153–155). Recently, Bolouri and colleagues demonstrated that IDR-1018 suppressed pro-inflammatory gene regulation in a neonatal mouse model of LPS-sensitized HI damage (156). The same group also suggested that post-HI treatment with IDR-1018 reduces LPS-induced HI brain damage. Therefore, IDRs might be promising neuroprotective agents for neonatal HI.

## Conclusion

Many neurodegenerative disorders have similar pathogenic mechanisms and data obtained from one disease may prove valid for another. There is a considerable amount of information in respect to the role of the C system, but there is no direct evidence for the involvement of AMPs in neonatal HI brain injury. Neonatal HI is associated with a robust inflammatory response, involving rapid change in neuronal gene expression associated with stimulation and aggregation of astrocytes and microglia for survival support (17). Activated microglia and astrocytes produce immunomodulatory proteins such as C components (73) and AMPs. We propose that misbalance of those proteins affects the equilibrium between pro- and anti-inflammatory cytokines in the CNS, resulting in prolonged inflammatory response and subsequent brain injury following neonatal HI. The regulation of the innate immune response simultaneously with cellular repair in the CNS is very complex, but the capability of both microglial and astroglial cells to produce C components and AMPs under cytokine stimulation suggests a role for both types of proteins in the brain. Whether this role is associated with the initiation or prolongation of the inflammatory response and subsequent damage following neonatal HI is unclear and needs further investigation. Deletion of some complement components such as C1q, as well as over-expression of others (C3a) has proven protective in neonatal HI brain damage. Application of CVF, C1-inhibitor, C3-inhibition through soluble CR1 or C3-deletion, as well as immunoglobulin treatment, have all shown protective effects in adult stroke animal models (157), suggesting that complement-targeted therapy could prove effective in neonatal HI and needs further investigation. The protective effects of IDRs in neonatal mouse models of HI suggest a potential key role for AMPs in the inflammatory response following neonatal HI brain injury. In conclusion, both C and AMPs appear to be key modulators of the innate and to some extent adaptive immune response following neonatal HI, which makes them potential candidates for neuroprotective strategy.

## Conflict of Interest Statement

The authors declare that the research was conducted in the absence of any commercial or financial relationships that could be construed as a potential conflict of interest.
